# Chitosan – Polyphosphate nanoparticles for a targeted drug release at the absorption membrane

**DOI:** 10.1016/j.heliyon.2022.e10577

**Published:** 2022-09-20

**Authors:** Ahmad Saleh, Zeynep Burcu Akkuş-Dağdeviren, Julian David Friedl, Patrick Knoll, Andreas Bernkop-Schnürch

**Affiliations:** a Center for Chemistry and Biomedicine, Department of Pharmaceutical Technology, Institute of Pharmacy, University of Innsbruck, Innrain 80/82, 6020 Innsbruck, Austria; b Department of Pharmacy, Universitas Mandala Waluya, A.H.Nasution, Kendari 93231, Southeast Sulawesi, Indonesia

**Keywords:** Chitosan, Polyphosphate, Polymeric nanoparticles, Drug delivery, Targeted release, Alkaline phosphatase

## Abstract

The aim of this study was to develop nanoparticles (NPs) providing a targeted drug release directly on the epithelium of the intestinal mucosa.

NPs were prepared via ionic gelation between cationic chitosan (Cs) and anionic polyphosphate (PP). The resulting NPs were characterized by their size, polydispersity index (PDI) and zeta potential. Isolated and cell-associated intestinal alkaline phosphatase (IAP) was employed to trigger polyphosphate cleavage in Cs-PP NPs which was quantified via malachite green assay. In parallel, the shift in zeta potential was determined. In-vitro drug release studies were performed in Franz diffusion cells with Cs-PP NPs containing rhodamine 123 as model active ingredient. Furthermore, cytotoxicity of Cs-PP NPs was assessed via resazurin assay on Caco-2 cells as well as via hemolysis assay on red blood cells.

Cs-PP NPs exhibited an average size of 144.17 ± 10.95 nm and zeta potential of -12.6 ± 0.50 mV. The encapsulation efficiency of rhodamine 123 by Cs-PP NPs was 86.8%. After incubation with isolated IAP for 3 h the polyphosphate of Cs-PP NPs was cleaved to monophosphate and zeta potential raised up to -2.3 ± 0.30 mV. Cs-PP NPs showed a non-toxic profile. Within 3 h, 62.0 ± 10.8% and 14.1 ± 2.2% of total rhodamine 123 was released from Cs-PP NPs upon incubation with isolated as well as porcine intestine derived intestinal alkaline phosphatase (IAP), respectively.

According to these results, Cs-PP NPs are promising drug delivery systems to enable a drug targeted release at the absorption membrane.

## Graphical abstract

**Figure:**
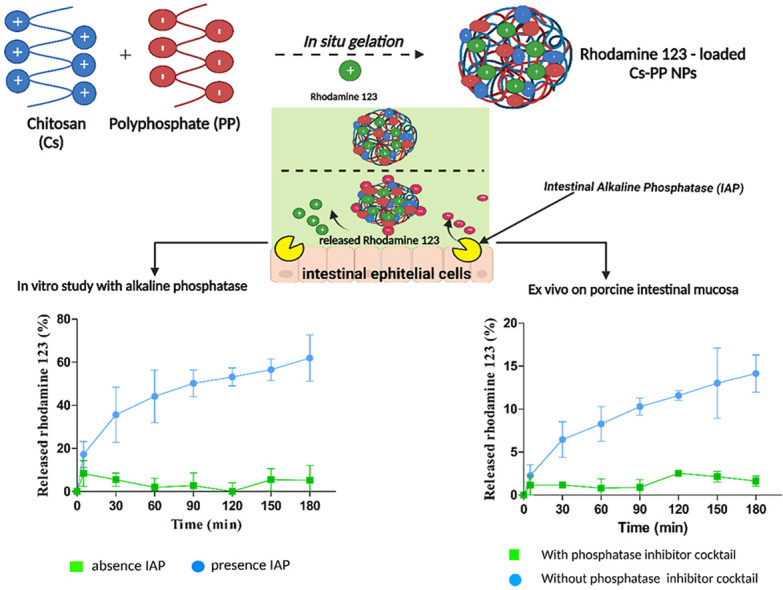

## 1 Introduction

As the oral route of administration is most convenient, the development of oral dosage forms is prioritized by the pharmaceutical industry. Numerous barriers of the gastrointestinal (GI) tract such as the harsh conditions in GI fluids, the mucus gel barrier and the absorption barrier, however, restrict the number of active pharmaceutical ingredients (APIs) that can be given orally. In order to overcome these barriers and to raise the number of orally administered drugs formulation scientists have pioneered diverse approaches such us enteric coatings, permeation enhancers or mucoadhesives [1, 2, 3, 4, 5, 6, 7].

A more recent strategy is focusing on a targeted release of APIs directly on the absorption membrane. It offers the advantages of high drug concentration directly at the absorption membrane representing the driving force for passive drug uptake. Furthermore, drugs that are inactivated under the harsh conditions of GI fluids can be shuttled to a much less aggressive environment of pH 7.2 and comparatively low enzymatic activity within the mucus gel layer close to the epithelium. The concept for such delivery systems is based on virus mimicking nanocarriers that are able to permeate the mucus gel layer because of a high density of anionic and cationic charges on their surface providing a targeted release of their payload directly at the epithelium [8, 9, 10]. This targeted release is triggered by the membrane-bound enzyme intestinal alkaline phosphatase (IAP) cleaving phosphate substructures on these nanocariers [5]. Although a proof of concept could already be provided [1, 2], this new technology is still in its infancy. Up to date just a targeted release of the macromolecular drug β-galactosidase could be shown [11]. Whether this concept might also work for small molecules, however, has so far not been investigated.

It was therefore the aim of this study to develop nanocarriers providing a targeted release of a small molecule directly at the absorption membrane of the small intestine. In contrast to previous studies, we take the advantage of a long chain polyphosphate (Graham's salt) consisting of 25 phosphate units forming nanocarriers with the cationic polysaccharide chitosan via ionic gelation. Rhodamine 123 was chosen as model small molecule for analytical reasons. The developed system was characterised concerning the cleavage of polyphosphate by IAP within the nanocarriers and the associated, shift in zeta potential, cytotoxicity and in vitro as well as ex-vivo release of rhodamine 123.

## 2 Material and methods

### 2.1 Materials

Chitosan (Cs) 25 kDa was purchased from Heppe Medical Chitosan GmbH (Halle, Germany) and sodium polyphosphate (PP) from Merck KGaA (Vienna, Austria). Alkaline phosphatase from bovine intestinal mucosa (7165 units/mg protein), ammonium molybdate tetrahydrate (8–83%), glucose-D-(+) ≥ 99.5% anhydrous, magnesium chloride anhydrous (MgCl_2_) ≥ 98%, minimum essential medium (MEM) eagle, malachite green oxalate salt 90%, potassium phosphate monobasic (KH_2_PO_4_) ≥ 99.5%, phosphatase inhibitor cocktail 2, resazurin sodium salt, rhodamine 123, Triton X-100, and anhydrous zinc chloride (ZnCl_2_) ≥ 97% were purchased from Sigma-Aldrich (Vienna, Austria). 4-(2-Hydroxyethyl)-1-piperazineethanesulfonic acid (HEPES) ≥ 99.5% was purchased from ROTH GmbH (Karlsruhe, Germany). Fetal bovine serum (FBS) and phosphate-buffered saline (PBS) were purchased from Biochrom, Merck (Tutzing, Germany). D-(+)-Trehalose dehydrate ≥98.0% was purchased from TCI Chemicals (Eschborn, Germany).

### 2.2 Preparation of Cs-PP NPs

Cs-PP NPs were prepared via in situ gelation between Cs as positively charged and PP as negatively charged polymer based on a previous described method with slight modifications [3, 8]. Cs was dissolved in 0.2% acetic acid at a concentration of 1 mg/ml under constant stirring for 24 h at 50 °C. After complete dissolution of Cs, pH was adjusted to 4.0 with 2 M sodium hydroxide solution. PP was dissolved in demineralized water at a concentration of 2.5 mg/ml and diluted with demineralised water for preparation of Cs-PP NPs in various Cs-PP mass to mass (m/m) as well as charge ratios as shown in Table 1. The charge ratio was calculated according to the following equation [12],

(1) $$Molar\;mixing\;ratio=\frac{F_{chitosan}.n_{chitosan}}{F_{PP}.n_{PP}}$$

where F and n represent the molar charge units and molar amount of anionic/cationic repeating units, respectively.

**Table 1:** Formulation and characterization of CS-PP NPs obtained by in situ gelation at indicated ratios.

Formulation	Cs–PP Ratio (m/m)	Cs–PP Charge Ratio	Cs Concentration (mg/ml)	PP Concentration (mg/ml)	Size (nm)	PDI	Zeta potential (mV)
F1	0.5	0.69	0.033	0.2	512.13 ± 13.47	0.423 ± 0.034	-3.52 ± 0.39
F2	0.25	0.35	0.033	0.4	345.10 ± 26.81	0.360 ± 0.012	-8.91 ± 1.31
F3	0.17	0.23	0.033	0.6	144.17 ± 10.95	0.284 ± 0.007	-12.6 ± 0.50

The polymer solutions were filtered through a 0.2 μm cellulose acetate filter (Sartorius AG, Gottingen, Germany). Then, 1 ml of PP was added dropwise into 3 ml of Cs solution (0.033 mg/ml) under constant magnetic stirring at 800 rpm and incubated for 30 min at 37 °C.

Suspensions of all developed Cs-PP NPs were centrifuged at 2500 g for 5 min with a MiniSpin centrifuge (Eppendorf, Hamburg, Germany) in order to purify the obtained Cs-PP NPs. To prevent the formation of aggregates 10 μl of 2% (m/v) trehalose was added to the Cs-PP NPs suspensions before centrifugation [5]. The supernatant was removed and NPs were resuspended in 1 ml of 100 mM HEPES buffer pH 7.5.

### 2.3 Encapsulation efficiency

In order to obtain rhodamine 123 loaded Cs-PP NPs 50 μl of rhodamine 123 (0.01% m/v in HEPES buffer pH 7.5) was added into 3 ml of CS solution that was prepared as described in section 2.2 and incubated at 800 rpm at room temperature for 30 min subsequent to the dropwise addition of 1 ml of PP solution that is prepared as described in section 2.2. Subsequently, rhodamine 123 loaded Cs-PP NP suspensions were purified by centrifugation. The supernatant was removed and NPs were suspended in 100 mM HEPES pH 7.5. Afterwards, 100 μl of rhodamine 123 loaded Cs-PP NPs suspensions were transferred to 96-well black plate and fluorescent intensity was measured (גex = 485 nm and גem = 535 nm) using a microplate reader (Tecan Infinite M200; Grödig, Austria). In order to quantify the amount of rhodamine 123 being entrapped in Cs-PP NPs, a calibration curve with increasing amounts of rhodamine 123 ranging from 0.78 to 50 μM was used. The percent of encapsulation efficiency (% EE) was calculated based on the following equation:

(2) $$\mathrm{\%EE}\;\text{=}\;\frac{Rhodamine\;123\;entrapped\;in\;Cs-PP\;NPs\;}{Initial\;amount\;of\;rhodamine\;123\;added}\times 100$$

### 2.4 Characterization of Cs-PP NPs

The polydispersity index (PDI), size (Z), and zeta potential (ζ-potential) of 0.01% (m/v) Cs-PP NPs were determined using a Zetasizer Nano ZS (Malvern Instruments, UK). Triplicated evaluations were carried out at 25 °C with a detection angle of 173°.

### 2.5 Enzymatic phosphate cleavage by isolated AP

Phosphate cleavage from Cs-PP NPs was examined by malachite green assay upon addition of isolated IAP [13]. In detail, to 10 ml of 0.01% (m/v) Cs-PP NPs in 100 mM HEPES buffer pH 7.5, 100 μl of IAP solution (10 U∖ml) was added and incubated at 37 °C under constant shaking at 300 rpm using a ThermoMixer (Eppendorf, Hamburg, Germany). Cs-PP NPs suspension without IAP addition represented the control and was incubated under equal conditions. Aliquots of 50 μl were transferred to a 96-well plate at predetermined time points (0, 30, 60, 90, 120, 150 and 180 min). To cease IAP activity during sampling and waiting times, 5 μl of 3.6 M H_2_SO_4_ was added to each well. Released monophosphate was measured through MLG assay. Accordingly, MLG salt was dissolved at a concentration of 0.15% (m/v) in 3.6 M H_2_SO_4._ Afterwards, 100 μl of Triton x-100 solution 11% (m/v) was added to 2.5 ml of MLG solution and incubated at 37 °C for 5 min. Thereafter. 1.5 ml of 8% (m/v) ammonium molybdate solution was added dropwisely under constant mixing by a vortex. To each test sample 100 μl of MLG reagent was added. Absorbance of resulting phospho-molybdate complexes was measured at a wavelength of 630 nm (Tecan Infinite M200; Grödig, Austria). The amount of released monophosphate was determined using a calibration curve with increasing amounts of KH_2_PO_4_ ranging from 0.78 to 50 μM.

### 2.6 Enzymatic phosphate cleavage on Caco-2 cells

Caco-2 cells were purchased from the European collection of cell cultures (ECACC, health protection agency, UK). Cells were cultured in 24 – well plates at a density of 25.000 cells/well with minimum essential medium (MEM) supplemented with 10% fetal bovine serum (FBS) and 1% penicillin-streptomycin at 37 °C and 5% CO_2_ atmosphere (95% relative humidity). The old MEM was changed on alternate days until a cell monolayer was observed. Prior to the experiment, cells were washed twice with 500 μl of glucose – HEPES buffer (268 mM glucose and 25 mM HEPES at pH 7.4). The control cells were incubated with 500 μl of glucose-HEPES buffer containing 1% (v/v) phosphatase inhibitor cocktail for 1 h prior to experiment. Afterwards, cells were washed again and incubated with 500 μl of 0.01% (m/v) Cs-PP NP suspension in glucose-HEPES buffer. Aliquots of 50 μl were transferred to 96 well plates at set time intervals (0, 30, 60, 90, 120, 150 and 180 min). As a control the experiment was performed under equivalent conditions with samples containing 1% (v/v) phosphatase inhibitor cocktail. Released monophosphate was evaluated via MLG assay as described in section 2.5.

### 2.7 Cell viability study

Caco-2 cells were cultivated as described above and washed twice with glucose–HEPES buffer prior to the experiment. Cs-PP NP suspensions were diluted in glucose–HEPES buffer pH 7.4 to obtain concentrations varying from 0.005% (m/v) to 0.01% (m/v). Thereafter, 500 μl of Cs-PP NPs were added to the wells and incubated at 37 °C for 2 h, 4 h and 24 h. Glucose – HEPES buffer pH 7.4 was utilized as negative control and 2% (m/v) Triton X-100 solution in glucose–HEPES buffer served as the positive control. At predetermined time points, cells were washed twice with 500 μl of the pre-warmed glucose–HEPES buffer. Afterwards, 250 μl of 2.2 mM resazurin solution were added to each well and incubated at 37 °C in dark conditions for 3 h. Then, 100 μL of aliquots from each well were transferred to a 96-well black plate and fluorescent intensity was measured at an excitation wavelength (גex = 540 nm) and an emission wavelength (גem = 590 nm) using a microplate reader (Tecan Infinite M200; Grödig, Austria).

Cell viability was evaluated by the following equation:

(3) $$\%Cell\;viability=\frac{Sample\;fluorescence}{negativ\;control\;fluorescence}\times 100$$

### 2.8 Hemolysis assay

Red blood cells (RBCs) were obtained form Tirol Kliniken GmbH (Zentralinstitute für Bluttransfusion und immunologische Abteilung) Innsbruck, Austria. RBCs were stored at 4 °C before use. Triton X-100 (1% m/v) was used as positive control and glucose-HEPES buffer pH 7.4 as negative control. RBCs were diluted 1/100 (v/v) in glucose-HEPES buffer pH 7.4. Cs-PP NPs (0.001%, 0.002%, 0.005% and 0.01% m/v) and RBCs were mixed in a ratio of 1/1 (v/v). In order to obtain de-phosphorylated Cs-PP NPs, 5 ml of Cs-PP NPs were incubated with 50 μl of IAP (10 U/ml) for 2 h under constant shaking at 300 rpm and 37 °C using a ThermoMixer (Eppendorf, Hamburg, Germany) prior to the experiment. Samples were incubated for 2 h, 4 h, 24 h or 48 h under constant shaking at 300 rpm and 37 °C using a ThermoMixer. Aliquots of 500 μl were centrifuged at 4617 g for 5 min at 20 °C. Thereafter, 100 μl of each supernatant were transferred to a 96 well plate and absorbance was recorded using a microplate reader at 415 nm (Tecan Infinite M200; Grödig, Austria). Percentage of lysis was calculated by the following equation:

(4) $$\;\text{\% ​​Lysis}\;\text{= ​​}\frac{\;Absorbance\;of\;sample\;in\;diluted\;RBC\;–\;Absorbance\;of\;HEPES\;buffer}{Absorbance\;of\;TritonX\;diluted\;RBC\;–\;Absorbance\;of\;HEPES\;buffer}\times 100$$

### 2.9 Drug release study by dialysis membrane

In vitro drug release from Cs-PP NPs was examined using a dialysis membrane method. Accordingly, 3 ml of rhodamine 123 loaded Cs-PP NPs containing 30 μl of IAP (10 U/ml) was poured in a dialysis bag (cellulose, MWCO: 14 kDa, Sigma Aldrich, Austria). Both ends were capped and the bag was submersed in a beaker containing 60 ml of 100 mM HEPES buffer pH 7.5. Rhodamine 123 loaded Cs-PP NPs suspension omitting IAP served as control and was incubated under equal conditions. Samples were incubated at 37 °C and 25 rpm in an orbital shaker (Orbital Shaker – Incubator ES-80, Grant Instruments Ltd., Cambridge SG8 6 GB, England) for 6 h. At predetermined time points aliquots of 100 μl were transferred in a 96 well black plate and replaced with the same volume of pre-warmed 100 mM HEPES buffer pH 7.5. Fluorescence intensity was measured at an excitation wavelength of 485 nm and an emission wavelength of 535 nm using a microplate reader (Tecan Infinite M200; Grödig, Austria). The released amount of rhodamine 123 was determined using a calibration curve as described in section 2.3.

The released rhodamine 123 from Cs-PP NPs was calculated as percent using the equation shown below:

(5) $$\%\;Released\;rhodamine\;123=\frac{Released\;rhodamine\;123}{Encapsulated\;rhodamine\;123\;in\;Cs-PPNPs}\times dilution\;factor\times 100$$

### 2.10 Drug release study on porcine intestinal mucosa

Studies on porcine intestinal mucosa were performed in Franz diffusion cells according to a previous described method [11]. Porcine small intestine was obtained from a regional abattoir and stored at -20 °C until future use. The intestine was cut into pieces and placed in the Franz diffusion cell with the mucus gel layer oriented to the top. Subsequently, 1 ml of rhodamine 123 loaded CS-PP NPs suspension of 0.01% (m/v) was filled into the donor compartment, while the acceptor chamber was filled with 4 ml of 100 mM HEPES buffer pH 7.5 and incubated at 37 °C under magnetic stirring. In parallel, samples were pre-incubated with 100 mM HEPES buffer pH 7.5 containing 1% (v/v) phosphatase inhibitor cocktail for 1 h under the same conditions serving as control. At predetermined time points aliquots of 400 μL were withdrawn through the sidearm and substituted with the same amount of pre-warmed 100 mM HEPES buffer pH 7.5. Aliquots were transferred into a 96 well black plate and fluorescence intensity was measured at גex = 485 nm and גem = 535 nm using a microplate reader (Tecan Infinite M200; Grödig, Austria). The released amount was determined as described above.

### 2.11 Statistical data analysis

The GraphPad Prism 5.01 software was used for statistical data analysis. All values are presented as means ± standard deviation (SD) (p ≤ 0.05). The levels of significance were set as the minimal level of significant (∗), very significant (∗∗) and for highly significant (∗∗∗) from three experiments. Differences between two independent groups were analysed by the unpaired student's t-test.

## 3 Results and discussion

### 3.1 Preparation and characterization of Cs-PP NPs

Cs-PP NPs were formed via ionic gelation. As size and zeta potential are key factors for these delivery systems, three different types of NPs were prepared by varying the mass ratio of positively charged Cs to negatively charged PP during the preparation process. By precise balancing the mass ratio of Cs and PP, defined polyelectrolyte complex particles in the nano-range were formed. The Cs-PP NP size range was between 144-512 nm as listed in Table 1. The more PP was added, the smaller was the particle size. As reported in various studies electrostatic interactions are the driving force for the formation of highly ionically cross-linked NPs. Kiilll et al., for instance, investigated the formation of Cs-PP polyelectrolyte NPs via multifactorial design and postulated that the more non-neutralized primary amino functions are available due to low PP concentration, the stronger is the intramolecular repulsion causing a stretching of the Cs chains and thus formation of large particles with pores [14].

Likewise, the zeta potential of NPs declined dependent on the mass ratio of Cs to PP. Surface charge decreased by more than Δ9 mV as a result of neutralization of the primary amino functions of Cs with polyanionic PP on the surface of NPs. In a previous study of Akkus et al. similarly a decrease in zeta potential, PDI and particles size was observed with increasing amounts of PP to branched polyethyleneimine (bPEI) forming PP-bPEI NPs [5]. As formulation F3 showed the highest homogeneity of the colloidal dispersion with a particle size of 144.15 ± 10.95 nm, polydispersity index of 0.284 and a zeta potential of -12.6 ± 0.50 mV it was chosen as most promising formulation for further studies.

### 3.2 Encapsulation efficiency (EE)

Rhodamine 123 is a hydrophilic small molecule having tertiary amine groups that make the compound a highly cationic fluorescence dye [15, 16, 17, 18]. Due to its charged nature and since it can be easily quantified by its fluorescent properties, it has been utilised as a tracer molecule and a model active ingredients to be incorporated into Cs-TPP NPs through its electrostatic interactions with the oppositely charged TPP [19, 20]. Furthermore, rhodamine 123 has been widely utilised as a p-glycoprotein (p-gp) substrate and therefore a model for the delivery of such substrates through biological membranes since p-gp efflux pumps are expressed in many biological barriers including the intestinal barrier [21]. Moreover, rhodamine 123 exhibits representative model properties for positively charged active pharmaceutical ingredients that need to be protected against anionic barriers on the way to the in target through the GI tract. Based on all these considerations rhodamine 123 was chosen as model active ingredient for release studies and encapsulated into Cs-PP NPs. In total 86.8% of rhodamine 123 was encapsulated in Cs-PP NPs. Results from a previous study by Essa et al. showed that rhodamine 123 can be successfully encapsulated in PEG-g-PLA nanoparticle with an EE between 10 and 68% depending on the type and properties of the polymer [17]. The comparatively high EE obtained in our study might be explained by the positive charge of rhodamine 123 interacting with negatively charged PP within the NPs.

### 3.3 Phosphate cleavage studies

#### 3.3.1 Enzymatic phosphate cleavage by isolated IAP

The catalytic enzyme IAP is located on the brush border membrane of the intestinal mucosa cleaving various biological domains such as phosphate esters of alcohols, amines and phenols [10, 22]. It is therefore the likely perfect target for site-specific drug delivery on the epithelium of the intestinal mucosa. In a previous study a time-dependent release of monophosphate from polyethyleneimine (PEI)-PP NPs was shown [5]. Le et al. observed also the elimination of PP from NPs due to its cleavage by IAP [23].

**Figure 1:**
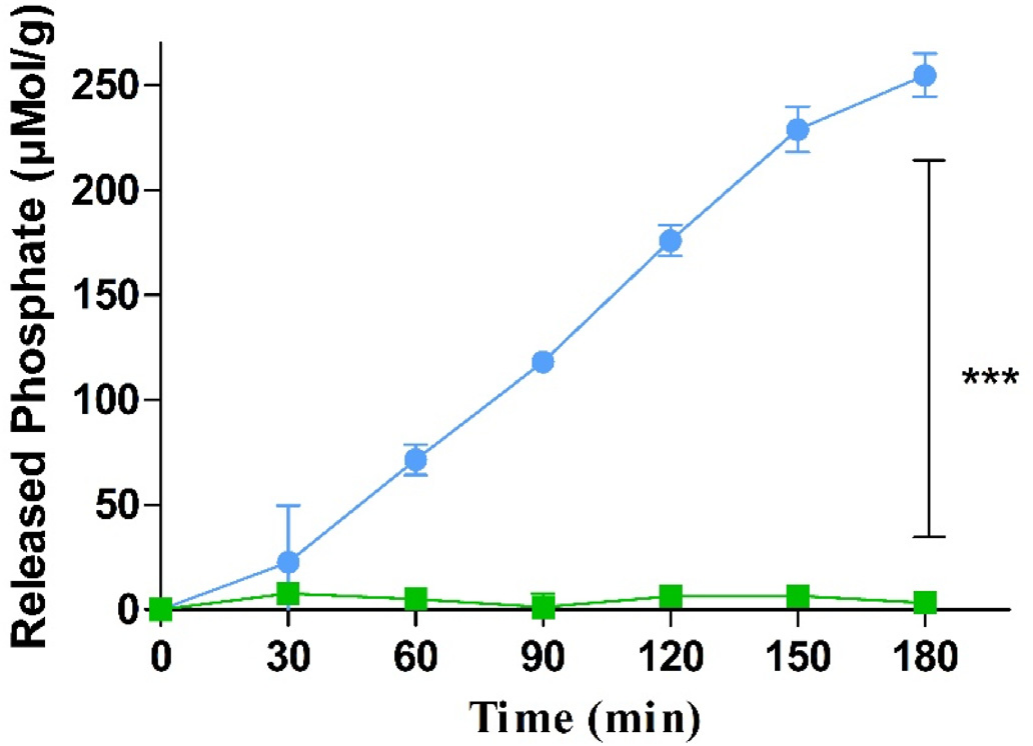
Phosphate release from CS-PP NPs in the presence () and absence () of isolated IAP (0.1 U/mL) at 37 °C. Data are indicated as means ± SD (n = 3). ∗∗∗ = p ≤ 0.001.

As illustrated in Figure 1 phosphate residues were rapidly cleaved from Cs-PP NPs by isolated IAP. In the absence of IAP, however, only a negligible amount of monophosphate was released.

The majority of IAP triggered phosphate releasing systems reported up to date such as micelles, self-emulsifying drug delivery systems (SEDDS), nanoemulsion and polymeric Cs NPs displayed a burst-release of monophosphate within the first 30 min. These results might be explained by the use of tripholyphosphate (TPP) for NPs formation. On the one hand, longer polyphosphate chains will contribute to a more stable ionic crosslinking and on the other hand the cleavage of these polyphosphates is more time consuming than that of TPP [11].

#### 3.3.2 Enzymatic phosphate cleavage by caco-2-cells

**Figure 2:**
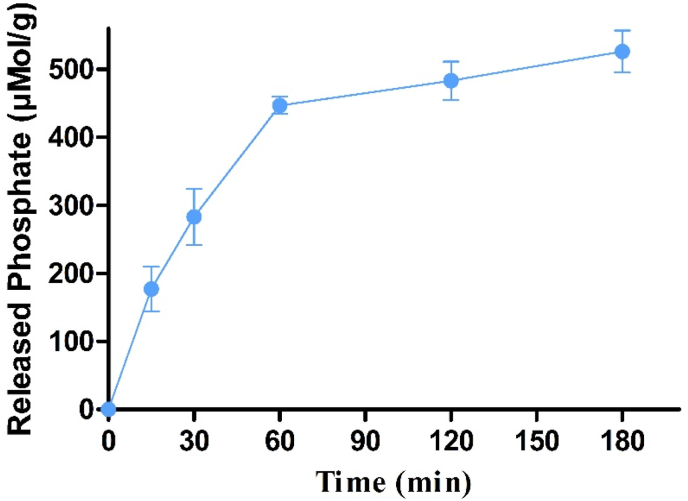
Phosphate release from Cs-PP NPs diluted in 268 mM glucose and 25 mM HEPES buffer pH 7.4 at 37 °C mediated by enzymatic cleavage with IAP expressed on Caco-2 cell monolayer. Data are indicated as means ± SD (n = 3).

To evaluate whether also membrane bound IAP can cleave phosphate from Cs-PP NPs a Caco-2-cell line was utilised. Incubation of Cs-PP NPs with Caco-2-cells resulted in a pronounced phosphate release within 3 h. As illustrated in Figure 2, even 526.16 μMol of phosphate were released within 180 min. According to these results Cs-PP NPs are even accessible for membrane bound IAP. These findings are in good agreement with previous studies demonstrating also a phosphate release from PEI-PP NPs on Caco-2-cells monolayer [5].

### 3.4 Enzyme induced shift in zeta potential

Exposure of Cs-PP NPs to IAP causes time-dependent cleavage of phosphate moieties from the particle surface. Due to the loss of negatively charged phosphate moieties, the zeta potential of the Cs-PP NPs should shift as a function of time. To confirm this assumption, Cs-PP NPs were incubated with IAP and the shift in zeta potential was monitored over 180 min.

**Figure 3:**
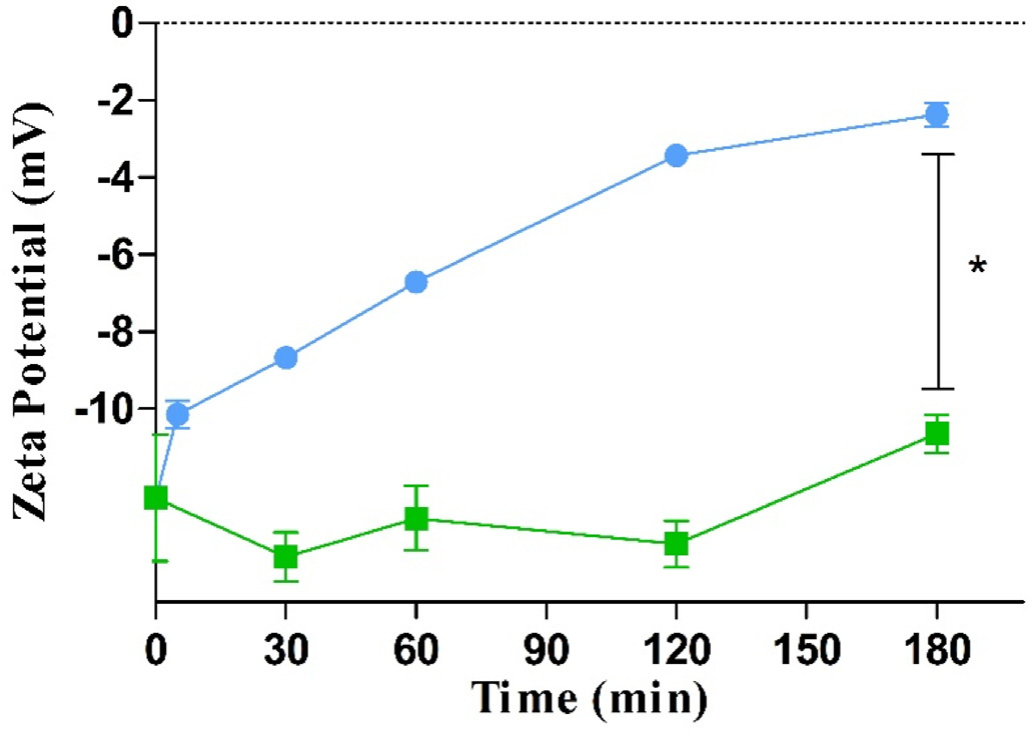
Time dependent ζ potential shift of CS-PP NPs in the presence () and absence () of isolated alkaline phosphate (0.1 U/mL) at 37 °C. Data are indicated as means ± SD (n = 3). ∗ = (p ≤ 0.05).

As illustrated in Figure 3 a continuous increase in zeta potential and a total shift of Δ 10.3 mV was observed within 180 min. After an initial rapid shift, the zeta potential raised continuously over the remaining observation time, which is in good correlation with results of phosphate cleavage. These results provide evidence that IAP efficiently cleaves phosphate groups from the surface of Cs-PP NPs causing a shift in zeta potential.

These results were in a good agreement the previous findings of Nazir et al. indicating also a shift in zeta potential of phosphorylated chitosan–chondroitin sulphate nanoparticles in the presence of IAP [3].

### 3.5 Cell viability assay

**Figure 4:**
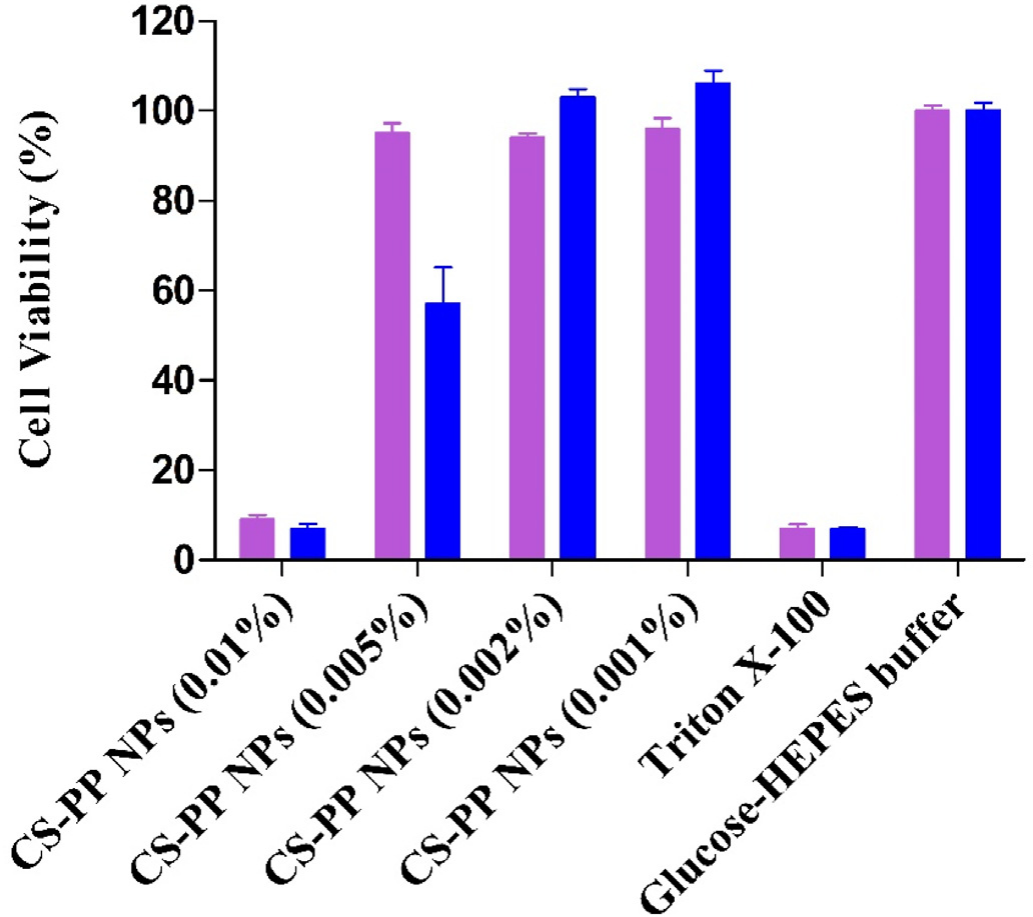
Cell viability of Caco-2 cells determined by resazurin assay after incubation with indicated concentrations of CS-PP NPs. Purple bars and blue bars depict % cell viability after incubation of cells with Cs-PP NPs for 2 and 24 h, respectively. Data are indicated as means ± SD (n = 3).

To evaluate cytotoxicity of Cs-PP NPs, a resazurin assay was performed on Caco-2 cells. Results displayed in Figure 4 depict a concentration dependent toxicity profile of Cs-PP NPs. At lower concentrations of 0.001% and 0.002% NPs did not show any toxicity but at higher concentrations cell viability was reduced significantly. Furthermore, at a concentration of 0.005%, a time-dependent toxicity could be noted since after 2 h cell viability was almost entirely provided whereas after 24 h just around 60% of cells survived.

The observed cytotoxicity can be explained by the cationic nature of chitosan. On one hand, its polycationic character enhances cellular interactions by associating with negatively charged cell membrane proteins and on the other hand membrane depolarisation can lead to membrane rupture or perturbation causing cellular toxicity [24, 25].

### 3.6 Hemolysis studies

**Figure 5:**
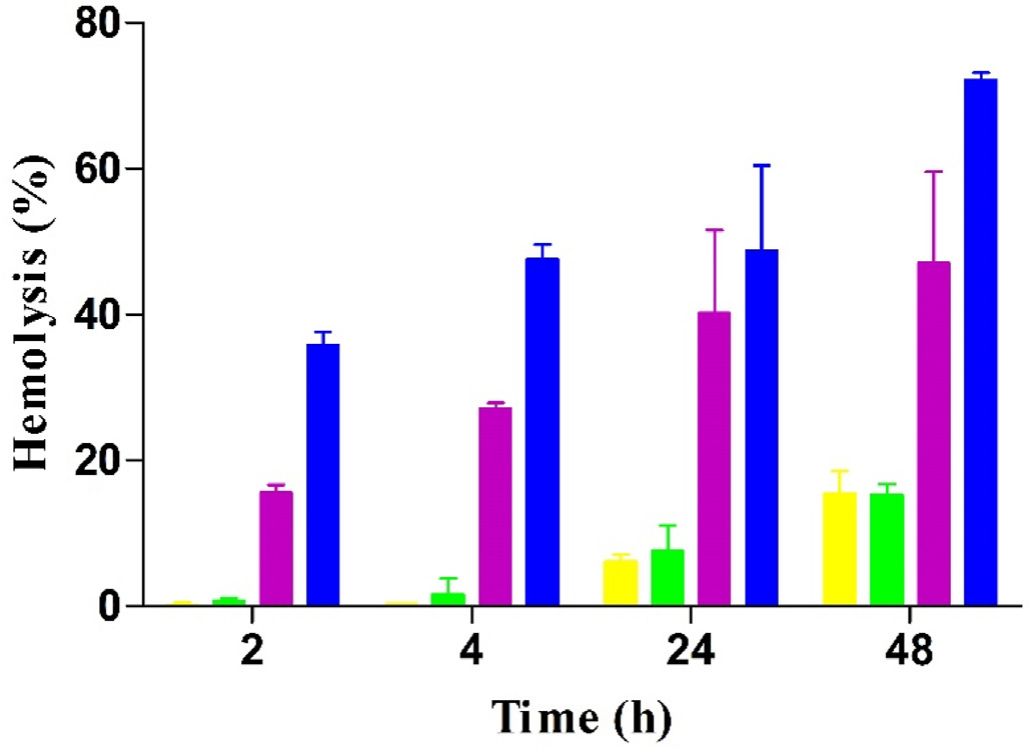
In vitro lysis of red blood cells caused by CS-PP NPs in increasing concentrations (0.001% m/v yellow bars, 0.005% m/v green bars, 0.002% m/v purple bars and 0.01% m/v blue bars) at indicated time points. Data are indicated as means ± SD (n = 3).

To evaluate cell membrane damage of Cs-PP NPs, hemolysis assay was performed on RBCs as their cell membrane is highly sensitive to cationic compounds and particles [26, 27, 28]. The safety profile of Cs-PP NPs in concentrations of 0.001%–0.01% is shown in Figure 5.

Results revealed that Cs-PP NPs are safe at lower concentrations independent from the incubation time. However, at higher concentrations, hemolysis activity of Cs-PP NPs increased depending on the incubation time. These results were in good agreement with a previous study of Sharifi et al. showing that the negative charge on the membrane of erythrocytes causes the release of hemoglobin via electrostatic interactions with the positively charged SEDDS droplets oily droplets of an O/*W nanoemulsions* [26].

### 3.7 Drug release studies

#### 3.7.1 Drug release studies with isolated IAP

**Figure 6:**
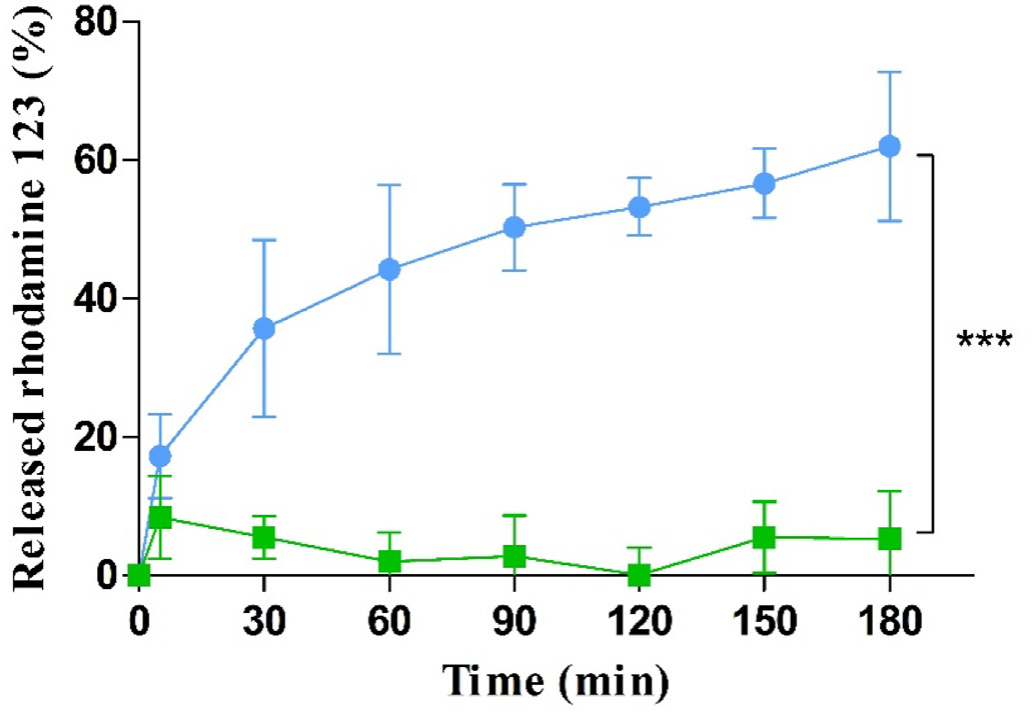
In vitro release of rhodamine 123 from Cs-PP NPs with () and without () IAP (0.1 U/mL) at 37 °C. Data are indicated as means ± SD (n = 3). ∗∗∗ = (p ≤ 0.001).

Ch-TPP NPs have been widely utilised in the field of drug delivery due to the strong ionic interactions between the oppositely charged Ch and TPP generating stable NPs that can encapsulate various APIs [29]. PP was recently introduced to the field of drug delivery as the only water soluble inorganic polyphosphate that is also able form NPs with Cs [14]. Similar to TPP, NP formation with PP occurs through ionic crosslinking of Cs with the polyanion generating a shell for APIs to be encapsulated. Accordingly, rhodamine 123 could be successfully encapsulated in Cs-PP NPs without being released unless PP was cleaved by IAP as shown in Figure 6.

Results showed a significant release of rhodamine 123 from Cs-PP NPs incubated with IAP. Within 180 min, 62.0% of rhodamine 123 was released from Cs-PP NPs. Due to its highly cationic nature and affinity to the hydrophilic polymers [15], rhodamine 123 release was negligible in the absence of IAP. Unlike particles incubated with IAP, the control samples omitting IAP displayed displaying no significant (p < 0.005) release. Haque et al. observed also a high affinity of rhodamine 123 to Cs-TPP NPs reaching an almost 90% encapsulation efficiency. Furthermore, just 28.6% of the rhodamine 123 were released from these NPs, unlike venlafaxine showing 44.3% release under the same conditions [30]. In another study mitomycin C and rhodamine 123 were encapsulated into Cs-PP NPs [19]. The release profile shown in Figure 6, can be explained by the loss of electrostatic interactions within Cs-PP NPs due to cleavage of PP by IAP in a time dependent manner destabilizing the network and causing the release of rhodamine 123 [31,32]. Accordingly, Cs-PP NPs might be utilised for the encapsulation and site-specific release of highly hydrophilic cationic APIs in general. In case of hydrophobic and uncharged APIs however, a burst release might be observed due to the low affinity of these compounds to the charged hydrophilic polymers.

Another parameter that might affect the release of rhodamine 123 from Cs-PP NPs is the encapsulation method. Ionic gelation is the most common and efficient encapsulation method for Cs NPs to protect a wide range of APIs from the production process for a proper release due to the lack of harsh conditions or usage of organic solvents unlike the alternative encapsulation methods [33]. However, due to the strong electrostatic interactions that enable the high encapsulation efficiency, the extent of the release of rhodamine 123 from the Cs-PP NPs was considerably low unless PP was cleaved by IAP.

#### 3.7.2 Drug release studies on porcine small intestinal mucosa

**Figure 7:**
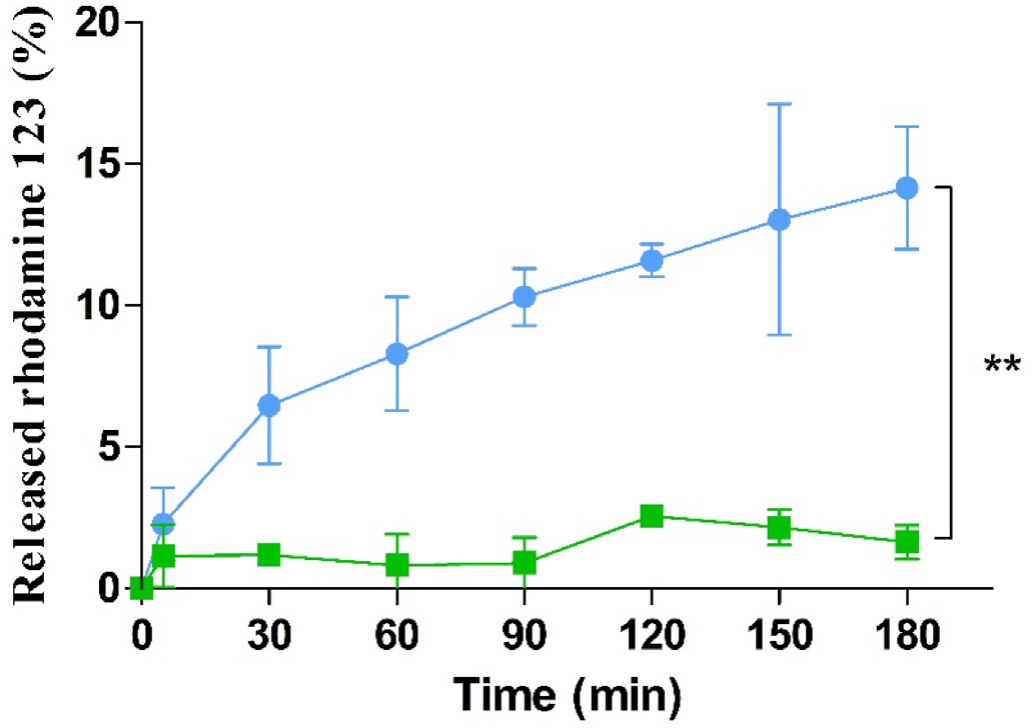
In vitro release of rhodamine 123 from Cs-PP NPs on porcine small intestinal mucosa at 37 °C in the absence () and presence of phosphatase inhibitor cocktail (). Data are indicated as means ± SD (n = 3). ∗∗ = (p ≤ 0.005).

To confirm results obtained with isolated IAP, release studies were also performed on intestinal mucosa as shown in Figure 7. Results provide evidence for the capability of Cs-PP NPs to cross the mucus barrier and to reach the intestinal epithelium, where PP is cleaved by IAP and rhodamine 123 is released. In contrast, control samples incubated with 1% (v/v) phosphatase inhibitor cocktail exhibited a negligible amount of rhodamine 123 being released. Negative surface charge of Cs-PP NPs facilitates the diffusion through the mucus gel layer providing a targeted release of rhodamine 123 at the intestinal epithelial membrane upon destabilisation of the particles by IAP. Similar results were obtained in a previous study by Leichner et al. reporting about the ability of Cs-TPP NPs to provide a targeted release of a protein at the epithelial layer of the small intestine by activation of the system upon contact with membrane-bound IAP [11]. In our study, we could demonstrate for the first time that such a targeted release can also be achieved for small molecules.

## 4 Conclusion

Within this study, Cs-PP NPs were developed for targeted drug delivery at the absorption membrane of the small intestine using IAP stimulation. Cs-PP NPs displayed a time-dependent phosphate cleavage upon contact with IAP enabling an enzyme triggered disintegration of the system causing the release of a model active ingredient. A prolonged release of rhodamine 123 was achieved due to usage of long polyphosphate chains (n = 25). Apart from the in vitro release studies with isolated enzyme, the release of rhodamine 123 from Cs-PP NPs was observed in an ex-vivo set-up using porcine small intestinal mucosa indicating that a targeted release takes can be provided in an environment similar to physiological conditions. This delivery system might be a useful tool in particular for oral delivery of active pharmaceutical ingredients that are poorly absorbed and that need to be released directly on the absorption membrane and protected from the harsh conditions of GI-fluids.

## Declarations

### Author contribution statement

Ahmad Saleh: Conceived and designed the experiments; Performed the experiments; Analyzed and interpreted the data; Wrote the paper.

Zeynep Burcu Akkuş-Dağdeviren; Julian David Friedl; Patrick Knoll: Performed the experiments; Analyzed and interpreted the data.

Andreas Bernkop-Schnürch: Conceived and designed the experiments; Contributed reagents, materials, analysis tools or data; Wrote the paper.

### Funding statement

This work was supported by the FWF (Fonds zur Fo¨rderung der wissenschaftlichen Forschung), Austria, under project number P 30268-B30. The authors would like to greatly acknowledge to The Ministry of Education, Culture, Research and Technology of The Republic of Indonesia for providing BPPLN scholarship scheme. Zeynep Burcu Akkuş-Dağdeviren and Julian Friedl were supported by a doctoral scholarship from Doktoratsstipendium aus dem Nachwuchsförderungs Programm of University of Innsbruck, Austria.

### Data availability statement

Data will be made available on request.

### Declaration of interest's statement

The authors declare no conflict of interest.

### Additional information

No additional information is available for this paper.
